# Arginine Methylation Antagonizes TEAD3‐Mediated Repression to Promote Osteogenic Differentiation by Disrupting RUNX2‐Sequestrating Condensates

**DOI:** 10.1002/advs.202518597

**Published:** 2026-01-20

**Authors:** Lei Cao, Ruohui Han, Hui Xiong, Qian Li, Shaofei Tao, Xudong Wu, Dayong Liu

**Affiliations:** ^1^ State Key Laboratory of Experimental Hematology The Province and Ministry Co‐sponsored Collaborative Innovation Center for Medical Epigenetics Key Laboratory of Immune Microenvironment and Disease (Ministry of Education) Tianjin Key Laboratory of Medical Epigenetics Department of Endodontics Tianjin Medical University School and Hospital of Stomatology & Tianjin Key Laboratory of Oral Soft and Hard Tissues Restoration and Regeneration Tianjin Medical University Tianjin China; ^2^ Department of Cell Biology Tianjin Medical University Tianjin China; ^3^ Department of Pediatric Dentistry Hospital of Stomatology and Hebei Provincial Key Laboratory of Stomatology Hebei Medical University Shijiazhuang China; ^4^ International Science and Technology Cooperation Base of Spinal Cord Injury Department of Orthopedic Surgery Tianjin Medical University General Hospital Tianjin China; ^5^ Shanghai Engineering Research Center of Tooth Restoration and Regeneration & Tongji Research Institute of Stomatology & Department of Endodontics Shanghai Tongji Stomatological Hospital and Dental School Tongji University Shanghai China; ^6^ Department of Physiology and Pathophysiology Tianjin Medical University Tianjin China

**Keywords:** arginine methylation, bone regeneration, epigenetics, osteogenic differentiation, periodontal ligament stem cells, TEAD

## Abstract

Osteogenic differentiation is essential for bone remodeling and repair. Protein arginine methyltransferases (PRMTs) regulate this process; however, their key substrates and mechanisms remain elusive. Here, we identify TEAD3, a TEA domain transcription factor mediating Hippo signaling output, as an arginine‐methylated regulator of periodontal ligament stem cells (PDLSCs) osteogenesis. Mechanistically, TEAD3 is methylated at arginine 55 (R55), a conserved residue within its DNA–binding TEA domain. Disruption of R55 methylation via R55K mutation enhances formation of TEAD3 homodimer condensates, which spatially constrain RUNX2 transcriptional activity without disrupting its Hippo signaling functions. Notably, the TEAD3‐R55K mutant exhibits heightened sensitivity to TEAi, a TEA domain‐ targeting inhibitory peptide. These findings unveil arginine methylation as a critical switch governing TEAD3‐mediated osteogenic commitment and highlight TEAD‐targeted strategies as promising therapeutics for bone regeneration.

## Graphical abstract

In the unmethylated state, TEAD forms stable, repressive condensates that sequester the osteogenic master regulator RUNX2. Arginine methylation of TEAD at R55 acts as a molecular brake, dissolving these condensates to release RUNX2 and activate the osteogenic program.

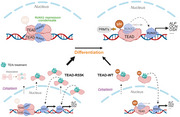

## 1 Introduction

Bone is a highly dynamic tissue that undergoes continuous remodeling to maintain structural integrity and respond to mechanical stress and injury [1, 2]. Bone remodeling involves the coordinated actions of osteoclasts, which resorb bone, and osteoblasts, which deposit new bone matrix. Osteogenic differentiation is essential for bone formation, contributing to both physiological bone remodeling and fracture healing [1, 3]. Gaining an in‐depth understanding of the regulatory mechanisms of osteogenic differentiation is of paramount importance for developing novel therapeutic approaches to treat various bone disorders, including osteoporosis, non‐union fractures, and bone defects.

Mesenchymal stem cells (MSCs) have demonstrated significant potential for clinical application in the treatment of diseases and tissue damage, primarily through their ability to govern the repair and regeneration of damaged tissues [4, 5, 6]. Periodontal ligament stem cells (PDLSCs), as a subset of tooth‐supportive tissue‐derived MSCs, possess superior abilities of direct differentiation and immune modulation. These abilities have been effectively harnessed in the maintenance and regeneration of bone tissues, as proved by both animal experiments and clinical trials [7, 8, 9, 10]. However, the regulatory mechanisms governing the osteogenic differentiation of PDLSCs remain incompletely elucidated, which poses a substantial barrier to their broader clinical application.

Runt‐related transcription factor 2 (RUNX2) is a key transcription factor in osteoblast differentiation and bone formation, primarily promoting osteoblast differentiation and mineralization by activating the expression of downstream genes [11, 12, 13]. It orchestrates the expression of osteoblast‐specific genes such as *Osteocalcin* (*OCN*), *Collagen type I* (*COL1A1*), and *Alkaline phosphatase* (*ALP*), while interacting with signaling pathways like BMP, Wnt, and FGF to finely balance osteoblast differentiation and mineralization [14, 15, 16, 17]. Despite its well‐established role, the dynamic regulatory mechanisms governing RUNX2 activity through post‐translational control of its interactome remain a central challenge of bone regenerative therapy. Within this context, the TEA domain transcription factors (TEADs), best known as terminal effectors of the Hippo signaling pathway governing cell expansion and organ size, have recently emerged as key RUNX2 regulators [18, 19, 20, 21]. The seminal study revealed that TEAD acts as a direct repressor of RUNX2 transcriptional activity, revealing a fundamental TEAD‐RUNX2 axis in osteoblast differentiation [22]. While this discovery fundamentally expanded the biological repertoire of TEADs beyond Hippo‐mediated growth control, critical questions persisted: How are TEAD‐RUNX2 interactions dynamically fine‐tuned by post‐translational modifications (PTMs) to respond to microenvironmental cues; Whether this non‐canonical TEAD activity could be therapeutically targeted without disrupting essential Hippo pathway functions. The absence of targeted molecular tools to precisely disrupt this interaction for therapeutic gain, relying instead on genetic manipulation of TEAD or its endogenous competitor VGLL4.

In this study, we resolve these questions by identifying arginine methylation as a Hippo‐independent mechanism that regulates the repressive function of TEAD3 in PDLSCs. We demonstrate that arginine methylation at TEAD3‐R55K mutation (arginine‐to‐lysine substitutions at positions 55) serves as a molecular switch that potentiates TEAD3 homopolymerization, thereby enhancing its inhibitory binding to Runt‐related transcription factor 2 (RUNX2) and suppressing osteogenesis. Capitalizing on this mechanism, we designed an inhibitory peptide (TEAi) that specifically targets the methylatable TEA domain to disrupt both TEAD3 self‐association and RUNX2 binding. This rationally designed intervention not only rescues osteogenic differentiation but achieves maximal efficacy under demethylated conditions (TEAD3‐R55K), validating its mechanism‐specific action. Our findings redefine TEAD as a dual‐function transcription factor that integrates Hippo signaling via YAP/TAZ while independently responding to post‐translational modifications to control RUNX2‐driven differentiation. By revealing this non‐canonical regulatory layer, we present a paradigm‐shifting strategy to modulate TEAD–RUNX2 activity for bone regeneration without compromising essential Hippo functions.

## 2 Results

### 2.1 TEAD Is Arginine Methylated During Osteogenic Differentiation of PDLSCs

To investigate the role of arginine methylation in osteogenic differentiation, we established an in vitro model using PDLSCs to induce osteogenic differentiation. Human PDLSCs were first isolated from healthy periodontal ligament tissues (Figure S1A). After a two‐week clonal expansion culture, cells exhibited high clonogenic potential, as evidenced by dense colony formation visualized through 0.5% crystal violet staining (Figure S1B). Flow cytometry analysis confirmed that the isolated PDLSCs present high surface markers for self‐renewal (CD90^+^/CD146^+^, >99%) and low levels of leukocyte common antigen (CD45, <1%), indicating a high purity (Figure S1C). Osteogenic commitment was quantitatively assessed by quantifying ALP activity (Figure S1D) and calcium deposition (Figure S1E), whereas adipogenesis was confirmed by lipid droplet accumulation using Oil Red O staining (Figure S1F). These assays collectively confirmed the suitability of the model for studying differentiation‐associated molecular mechanisms.

**FIGURE 1:**
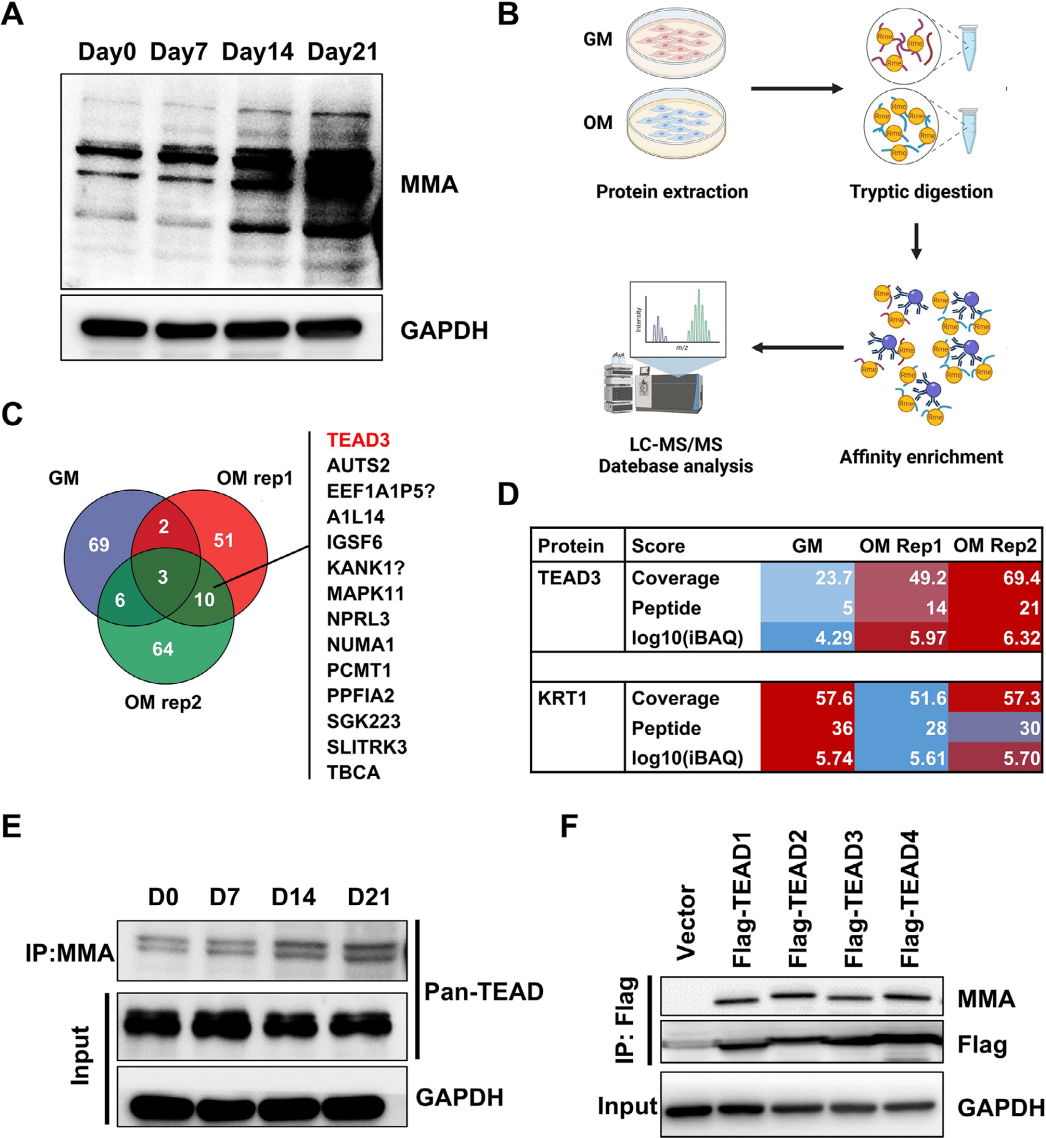
TEAD is arginine methylated during osteogenic differentiation in PDLSCs. (A) Western Blot analysis of MMA during osteogenic differentiation of PDLSCs. (B) Processes to identified arginine methylated protein during osteogenic differentiation. (C) Screen for proteins with identified arginine methylated peptides during osteogenic differentiation. (D) MS analysis of TEAD3 and KRT1. (E) Co‐IP was performed by MMA antibody and the pull downed proteins were subjected to Western Blot. (F) Flag‐tagged TEAD1‐4 were transfected into 293T cells, immunoprecipitated using a Flag‐specific antibody, and subsequently analyzed by Western blot.

To explore the dynamics of arginine methylation during osteogenic differentiation, we first monitored global mono‐methyl arginine (MMA) using a specific monoclonal antibody. Intriguingly, endogenous MMA signals exhibited a time‐dependent increase throughout osteogenic differentiation (Figure 1A). While this finding indicated a potential role for arginine methylation in osteogenesis, it did not identify the specific proteins modified by MMA that could be functionally important. To pinpoint these key effector proteins, we performed immunoprecipitation (IP) of MMA‐modified proteins followed by mass‐spectrometry (MS) (Figure 1B). This proteomic screening identified ten candidate proteins harboring arginine‐methylated peptide(s) (Figure 1C). Among these, TEAD3 was prioritized for further investigation based on two criteria (Figure 1D): (1) its known role as a transcriptional repressor of RUNX2 [22]. And (2) its presence in multiple differentiation‐associated signaling pathways [23, 24]. Subsequent validation experiments confirmed that Pan‐TEAD proteins demonstrated progressively increasing MMA levels during osteogenic differentiation (Figure 1E). Furthermore, ectopic expression of Flag‐tagged TEAD1‐4 demonstrated that all four TEAD family proteins could be susceptible to arginine methylation, as detected by MMA‐specific antibody (Figure 1F). These findings suggest that TEAD may be involved in regulating osteogenic processes through arginine methylation.

### 2.2 TEAD3 is Methylated at Arginine 55 by Multiple Methyltransferases

**FIGURE 2:**
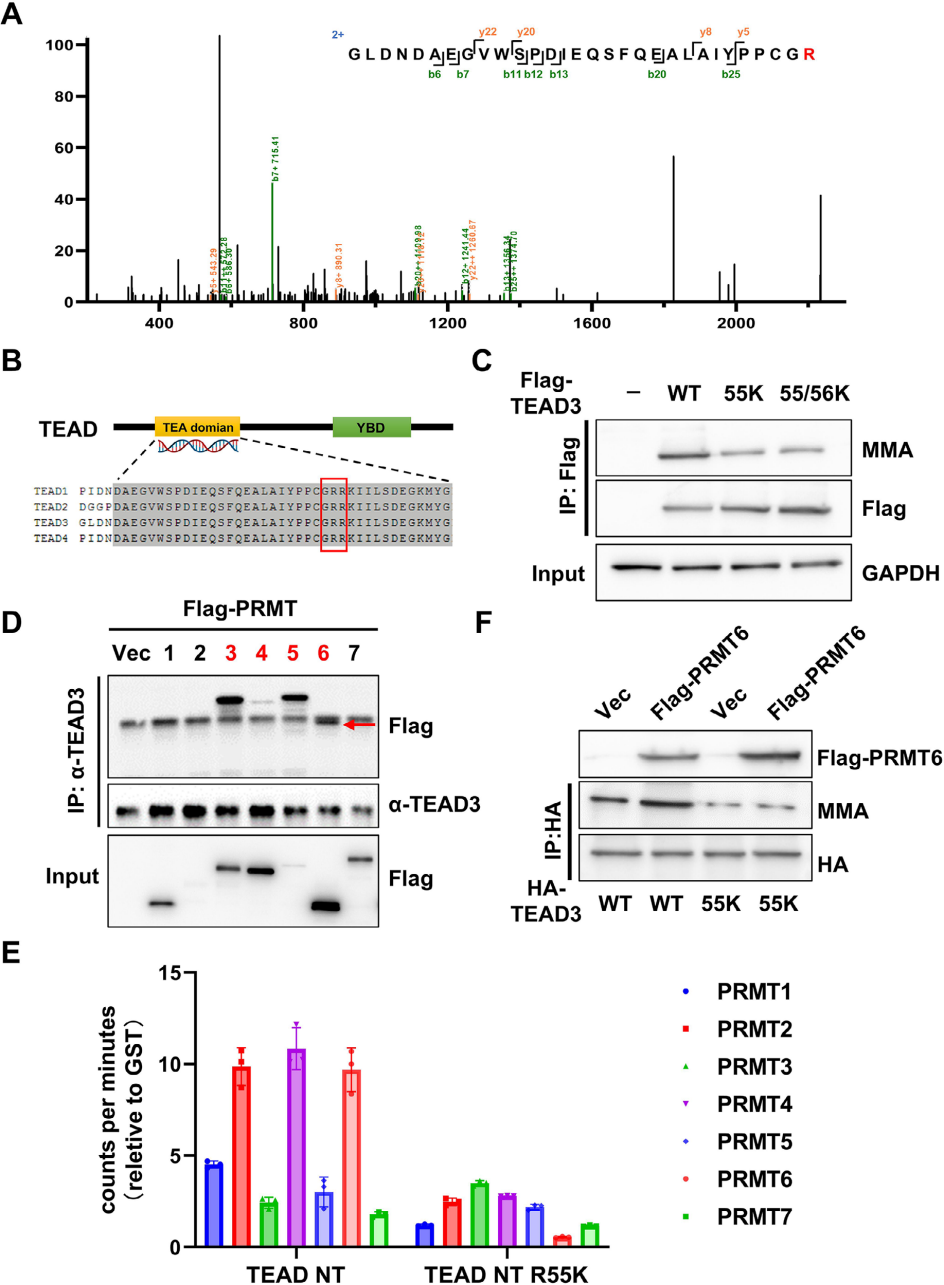
TEAD3 is methylated at arginine 55 by multiple methyltransferases. (A) Methylated peptide of TEAD3 was identified by MS. (B) Sequence alignment of the putative methylated site of TEAD. (C) WB analysis was performed to compare the MMA levels of Flag‐tagged TEAD3 and its mutants. (D) Interaction between TEAD3 and PRMT1‐7. (E) In vitro methylation assays revealed that PRMT2, PRMT4 and PRMT6 methylated the N‐terminal domain of TEAD WT but not the R55K mutant. (F) WB analysis was performed to compare the MMA levels of indicated groups.

Mass spectrometry analysis of MMA‐modified proteins identified arginine 55 (R55) within TEAD3 as a unique methylation site (Figure 2A). This residue is located within a conserved GRR motif (residues 54–56) of the TEA DNA binding domain, which is a preferred substrate motif of type I PRMT [25] (Figure 2B). To validate R55 methylation in cultured cells, we overexpressed Flag‐tagged wild‐type TEAD3 (WT) or mutants (R55K and R56K, arginine‐to‐lysine substitutions at positions 55 and 56, respectively) in 293T cells. Lysine substitutions retain the positive charge of arginine but preclude methylation, enabling functional assessment of site‐specific modifications [26]. Strikingly, the R55K mutation but not R56K significantly diminished MMA signals (Figure 2C), confirming that R55 is the primary, though not sole, arginine methylation site. To identify the methyltransferase(s) responsible for R55 methylation, we screened a panel of arginine methyltransferases (PRMTs), including PRMT1‐7, for physical interaction with TEAD3. Co‐immunoprecipitation (Co‐IP) assays revealed specific binding of PRMT3, PRMT4, PRMT5, and PRMT6 to TEAD3 (Figure 2D). Subsequent in vitro methylation assays using the N‐terminal domain of TEAD3 (harboring the GRR motif) demonstrated that PRMT2, PRMT4, and PRMT6 catalyzed methylation of TEAD WT but not the R55K mutant (Figure 2E). Notably, PRMT6 overexpression markedly enhanced TEAD3 arginine methylation, while showing no effect on the R55K mutant (Figure 2F). The potent activity of PRMT6 in overexpression assays prompted us to investigate its necessity under physiological conditions. Surprisingly, short hairpin RNA (shRNA)‐mediated knockdown of PRMT3, PRMT4, or PRMT6 individually, failed to significantly reduce the mono‐methylarginine (MMA) level of endogenous TEAD3 in PDLSCs (Figure S2A, B). This result suggested potential functional redundancy among these enzymes. We therefore performed combinatorial knockdowns. Strikingly, a marked reduction in TEAD3 methylation was observed only upon the simultaneous depletion of PRMT3, PRMT4, and PRMT6 (Figure S2A, B). However, this triple knockdown also induced severe cytotoxicity (Figure S2C), preventing a meaningful assessment of its functional consequences on osteogenesis. Collectively, these data demonstrated that TEAD3 is predominantly arginine‐methylated at R55 and suggested that multiple PRMTs may cooperate for R55 methylation, which warrants further investigation.

### 2.3 R55K Mutation Enhances the Repressive Capability of TEAD3 on Osteogenic Differentiation

**FIGURE 3:**
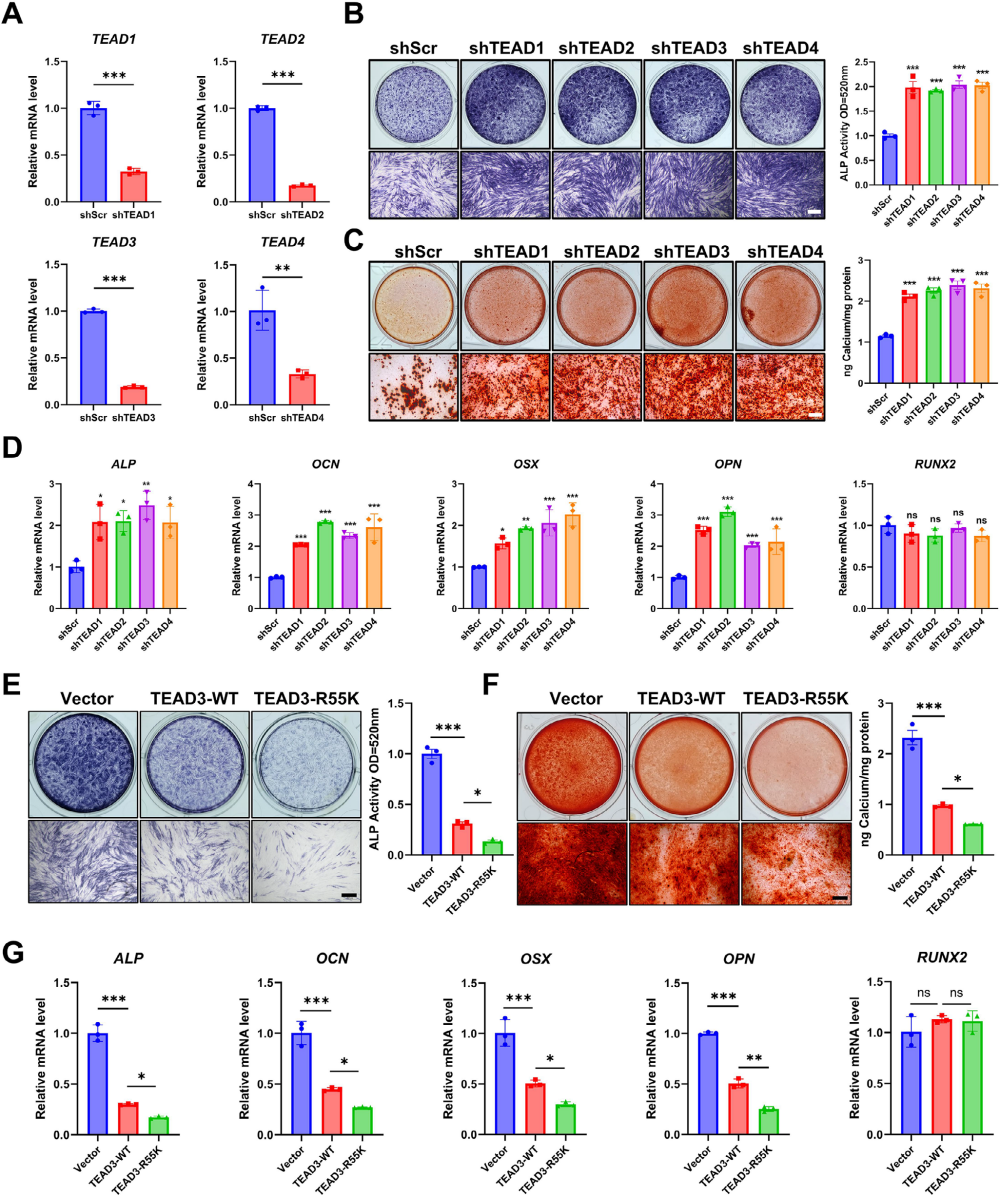
R55K mutation enhances the repressive capability of TEAD3 on osteogenic differentiation. (A) Knockdown efficiency of TEAD in PDLSCs was measured by RT‐qPCR. shScr indicats the scramble short hairpin RNA as a control. ^**^ *P* < 0.01, ^***^ *P* < 0.001. (B) ALP staining and ALP activity quantification at 7 days of osteogenic differentiation. Scale bar, 200 µm. (C) Alizarin red staining and quantitative analysis of calcium ion concentration at 14 days of osteogenic differentiation. Scale bar, 200 µm. (D) Relative mRNA levels of osteogenic marker genes were quantified by RT‐qPCR at 7 days of osteogenic differentiation. ^*^ *P* < 0.05, ^**^ *P* < 0.01, ^***^ *P* < 0.001, ns: *P* > 0.05 vs. shScr. (E)‐(G) PDLSCs were infected with TEAD‐WT and TEAD‐R55K lentivirus respectively. Osteogenic differentiation was evaluated by ALP staining and ALP activity quantification at 7 days (E), Alizarin red staining and quantitative analysis of calcium ion concentration at 14 days (F). Scale bar, 200 µm. Relative mRNA levels of osteogenic marker genes were quantified by RT‐qPCR (G). ^*^ *P* < 0.05, ^**^ *P* < 0.01, ^***^ *P* < 0.001, ns: *P* > 0.05. All data are expressed as means ± SD (n = 3).

To elucidate the role of TEAD transcription factors in regulating osteogenic differentiation of PDLSCs, we systematically manipulated TEAD expression using loss‐ and gain‐of‐function approaches. First, short hairpin RNA (shRNA)‐mediated knockdown targeting TEAD3 and its paralogs was performed to assess functional redundancy within the TEAD family. RT‐qPCR confirmed efficient reduction of all four TEAD isoforms at the mRNA level (Figure 3A). Silencing TEADs significantly enhanced osteogenic differentiation of PDLSCs, evidenced by increased ALP activity, accelerated calcium nodule formation (Figure 3B, C), and upregulated expression of osteogenic markers (*ALP*, *OCN*, *OSX*, and *OPN*) compared to scramble controls (Scr), while the mRNA levels of *RUNX2* were not affected (Figure 3D). These results strongly suggested that TEAD family members collectively act as negative regulators of osteogenesis.

To corroborate these findings and exclude confounding effects from potential TEAD‐mediated expansion changes during differentiation, we employed a doxycycline‐induced Tet‐on system for tightly controlled overexpression of individual TEAD isoforms (TEAD1‐4). Immunofluorescence (IF), RT‐qPCR, and Western blotting (WB) analyses confirmed nuclear‐localized overexpression of TEADs (Figure S3A–C). Strikingly, TEAD overexpression suppressed osteogenic differentiation, evidenced by diminished ALP staining, reduced mineralization, and downregulation of osteogenic markers, mirroring but opposing the knockdown phenotype (Figure S3D–F).

Given the observed repressive role of TEADs, we next investigate whether R55 methylation modulates this regulatory function. Using the Tet‐on system, we further overexpressed TEAD3‐R55K mutants to mimic the unmethylated state. Nuclear localization and expression levels of TEAD3‐R55K were comparable to wild‐type (WT) TEAD3 (Figure S4A–C). Intriguingly, TEAD3‐R55K exhibited enhanced repressive activity, further suppressing ALP activity and mineralization (Figure 3E, F), while exacerbating the downregulation of osteogenic markers (Figure 3G). This hyper‐repressive phenotype suggests that R55 methylation normally attenuates TEAD3's inhibitory function, potentially through structural or co‐factor recruitment changes.

### 2.4 R55K Promotes RUNX2 Sequestration Through Enhanced Homodimerization

**FIGURE 4:**
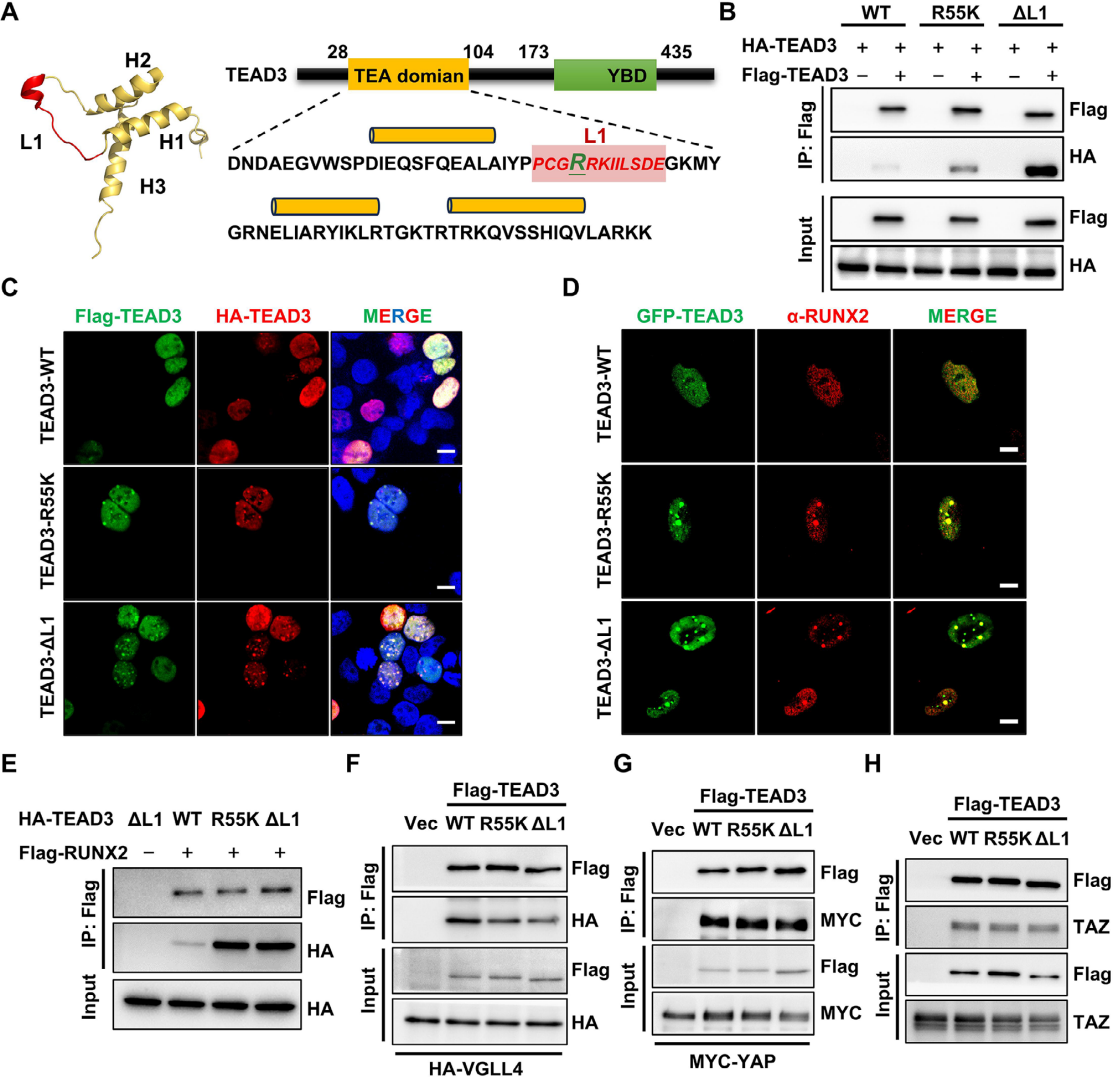
R55K promotes RUNX2 sequestration through enhanced homodimerization. (A) The 3D solution NMR structure of TEAD DBD (PDB id: 2HZD). The L1 loop is shown in red. Amino acid sequence of the DNA‐binding domain of the human TEAD3 transcription enhancer factor. Amino acids deleted in the ΔL1 mutant are shown in red. (B) Detection of TEAD3 homodimer formation by Co‐IP. (C) The colocalization of Flag‐TEAD3 and HA‐TEAD3 was showed by IF. Scale bar, 10 µm. (D) The colocalization of TEAD3 condensates with RUNX2. Scale bar, 10 µm. (E)‐(H) Detection of the interaction of TEAD3 WT and R55K mutation with RUNX2 (E), VGLL4 (F), YAP (G) and TAZ (H) by Co‐IP respectively.

The X‐ray crystallographic structure of TEA domain reveals that R55 resides within a flexible 12‐amino acid linker (L1) connecting helix 1 and helix2 (Figure 4A). Intriguingly, a previously reported L1 deletion mutant (ΔL1) adopts a helix‐swapped homodimer formation, where helix 1 is exchanged between monomers [27], suggesting that L1 flexibility modulates dimerization dynamics. Our functional studies demonstrated that both the ΔL1 mutation and the R55K substitution, which mimics constitutive demethylation, not only enhanced the suppression of osteogenic differentiation but also induced a pronounced further reduction in the expression of osteogenesis‐associated markers (Figure S5). These observations led us to hypothesize that R55 methylation regulates TEAD dimerization propensity, thereby influencing its transcriptional repressive activity.

To test this hypothesis, we first assessed homodimerization of HA‐ and Flag‐tagged TEAD3 in HEK293T cells. Wild‐type TEAD3 exhibited minimal self‐interaction, whereas both the R55K mutant and ΔL1 mutant robustly promoted dimerization (Figure 4B). IF analysis further revealed that R55K and ΔL1 mutants formed nuclear condensates enriched with self‐associated TEAD3 (Figure 4C), consistent with enhanced homodimerization or oligomerization.

RUNX2, a master transcriptional activator of osteogenesis, binds both the N‐ and C‐terminal regions of TEAD [22]. During the osteogenic differentiation, VGLL4 displaces RUNX2 from TEAD by competing for binding, thereby relieving TEAD‐mediated repression [22]. We thus posited that R55K/ΔL1‐induced condensates might aberrantly sequester RUNX2. Strikingly, endogenous RUNX2 extensively colocalized with R55K and ΔL1 condensates but showed minimal association with wild‐type TEAD3 (Figure 4D). Co‐IP assays confirmed that R55K strengthened TEAD3‐RUNX2 interaction while diminishing TEAD3‐VGLL4 binding (Figure 4E, F). This shift in binding partners likely underlies the enhanced repression of osteogenesis, as persistent RUNX2 sequestration would block its transcriptional activation of osteogenic genes. Importantly, the R55K mutation did not alter TEAD3's interaction with YAP/TAZ, the canonical Hippo pathway coactivators (Figure 4G, H), demonstrating that R55 methylation specifically modulates a non‐canonical regulatory axis distinct from YAP/TAZ signaling.

### 2.5 R55K Mutation Enhances Susceptibility to Inhibition by the TEA Domain‐specific Targeting Peptide in Vitro

**FIGURE 5:**
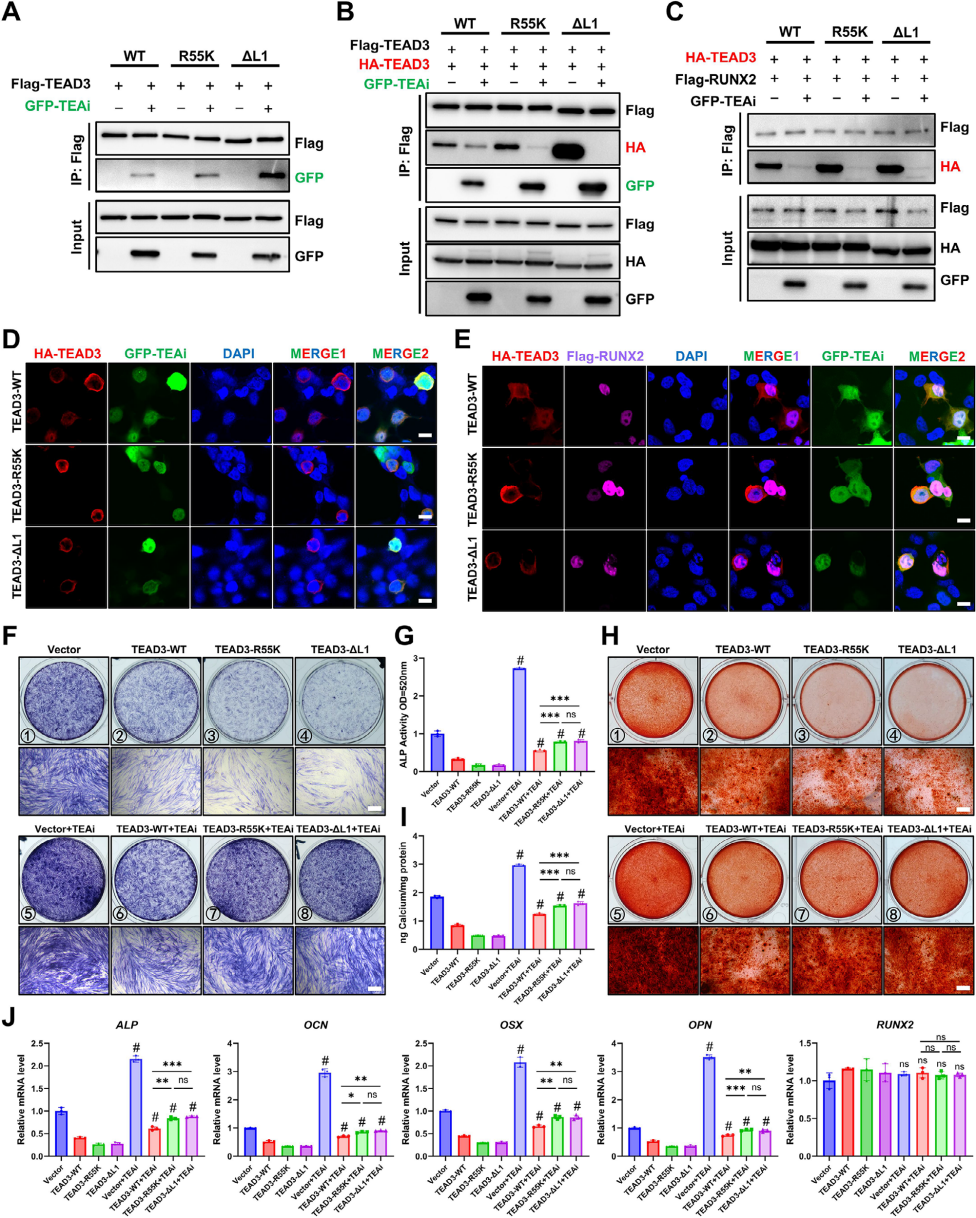
R55K mutation enhances susceptibility to inhibition by the TEA domain‐specific targeting peptide in vitro. (A) TEAi could interact with TEAD3 WT, R55K and ΔL1 mutation. (B) Co‐IP was performed to determine the effect of TEAi on TEAD homodimer formation. (C) Co‐IP was performed to determine the effect of TEAi on TEAD‐RUNX2 interaction. (D) IF analysis was used to determine the effect of TEAi on TEAD homodimer condensates. Scale bar, 10 µm. (E) IF analysis was used to determine the effect of TEAi on subcellular localization of TEAD3 and RUNX2. Scale bar, 10 µm. (F)‐(J) Effect of TEAi on osteogenic differentiation of PDLSCs in vitro was evaluated by ALP staining and ALP activity quantification at 7 days (F‐G), Alizarin red staining and quantitative analysis of calcium ion concentration at 14 days (H‐I). Scale bar, 200 µm. Relative mRNA levels of osteogenic marker genes were quantified by RT‐qPCR (J). ^#^ *P* < 0.05 vs. corresponding control group without TEAi, ^*^ *P* < 0.05, ^**^ *P* < 0.01, ^***^ *P* < 0.001, ns: *P* > 0.05. All data are expressed as means ± SD (n = 3).

Our findings establish that the TEA domain of TEAD, particularly residue R55, serves as a critical structural determinant for mediating TEAD‐RUNX2 interactions. This observation aligns with prior reports demonstrating that the isolated TEA domain alone is sufficient to bind RUNX2 [22], underscoring its central role in forming inhibitory complexes. Building on this, we hypothesized that pharmacologically disrupting the TEA domain could liberate RUNX2 from TEAD‐mediated sequestration, thereby reactivating osteogenic programs. To test this, we utilized a *Drosophila*‐derived inhibitory peptide, TEAi, engineered to competitively bind the TEA domain [28]. For all experiments involving TEAi, we utilized intracellular transgenic expression of a construct in which the TEAi peptide was fused in‐frame to the N‐terminus of GFP. Co‐IP assays confirmed that TEAi directly interacts with TEAD3 and potently suppresses its homodimerization (Figure 5A, B). Intriguingly, TEAD3‐R55K and ΔL1 mutants, both exhibiting enhanced dimerization propensity (Figure 5B), displayed stronger TEAi binding than wild‐type TEAD3 (Figure 5A), suggesting that structural destabilization of the TEA domain (via methylation loss or L1 deletion) increases the accessibility for TEAi engagement.

We next evaluated TEAi's ability to disrupt TEAD‐RUNX2 complexes. Notably, TEAi treatment nearly abolished TEAD3‐RUNX2 interactions across wild‐type, R55K, and ΔL1 mutants (Figure 5C), indicating robust and mutation‐agnostic inhibitory activity. Furthermore, TEAi expression effectively dissolved TEAD3 condensates in all tested groups, as visualized by IF, and unexpectedly promoted TEAD3 nuclear export, a phenomenon warranting further mechanistic investigation (Figure 5D and Figure S6A). A tricolor fluorescence assay confirmed that TEAi fully rescued RUNX2 from TEAD3 condensates without altering RUNX2's nuclear localization, thereby restoring its transcriptional availability (Figure 5E and Figure S6B).

To assess TEAi's functional impact, we treated PDLSCs with TEAi by a transgenic approach during osteogenic induction. TEAi significantly enhanced osteogenic differentiation, as evidenced by elevated ALP activity, accelerated calcium mineralization, and upregulated expression of osteogenic markers (Figure 5F–J). Notably, while R55K and ΔL1 mutants exhibited stronger baseline suppression of differentiation compared to wild‐type TEAD3, they were paradoxically more sensitive to TEAi‐mediated rescue (Figure 5F–J). This inverse correlation between dimerization propensity and TEAi efficacy suggests that hyper‐stable TEAD condensates are more vulnerable to targeted disruption.

Collectively, these data position the TEA domain as a druggable interface for modulating TEAD‐RUNX2 interactions. The enhanced susceptibility of methylation‐deficient mutants (R55K/ΔL1) to TEAi implies that pathological TEAD hyper‐dimerization, driven by loss of arginine methylation, creates a therapeutically exploitable vulnerability. Our findings propose a dual‐path strategy: (1) direct TEA domain targeting with TEAi to dismantle repressive condensates, and (2) adjunctive modulation of R55 methylation to amplify TEAi's therapeutic window.

### 2.6 TEAi Enhances TEAD3‐Dependent Bone Regeneration in a Rat Calvarial Defect Model

**FIGURE 6:**
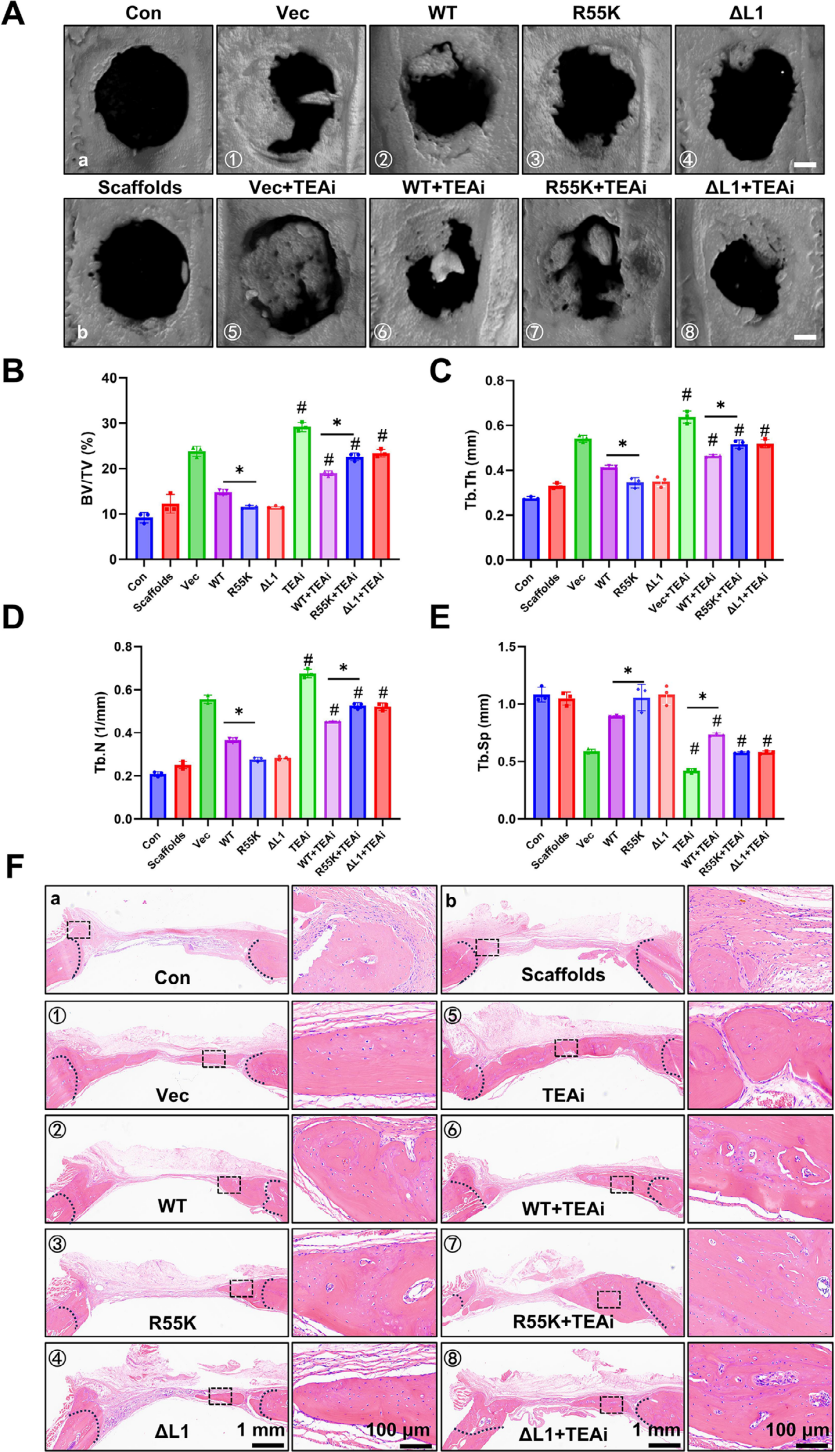
Effect of TEAi on bone remodeling of PDLSCs in calvarial bone defect model. (A) The images of Micro‐CT reconstruction of calvarial bone. Scale bar: 1 mm. (B‐E) Statistical analysis: BV/TV (B), Bone volume/Tissue volume. Tb.Th (C), Trabecular thickness. Tb.N (D), Trabecular numbe. Tb.Sp (E), Trabecular separation. ^#^ *P* < 0.05 vs. corresponding control group without TEAi. ^*^ *P* < 0.05. All data are expressed as means ± SD (n = 3). (F) The new bone formation ability in bone defect areas was detected by HE staining.

To evaluate the therapeutic potential of TEAD3‐R55 methylation modulation and TEAi in bone regeneration, we employed a well‐established rat calvarial defect model [29]. Bilateral full‐thickness bone defects (5 mm diameter) were surgically created, and defects were implanted with PDLSCs embedded in Pluronic F‐127/gelatin sponge scaffolds to mimic clinical bone repair strategies (Figure S7A). At 12 weeks post‐implantation, micro‐CT scans and 3D reconstructions revealed that the expression of TEAi robustly enhanced bone regeneration, with experimental groups (⑤‐⑧) showing significantly greater new bone volume compared to non‐TEAi controls (①‐④) (Figure 6A). Quantitative morphometric analysis demonstrated that TEAi groups exhibited elevated bone volume fraction (Bone volume/Tissue volume, BV/TV), increased trabecular thickness (Tb.Th) and trabecular number (Tb.N), reduced trabecular separation (Tb.Sp), collectively indicating superior bone microarchitecture (Figure 6B–E). Histological validation via hematoxylin‐eosin (HE) staining confirmed that TEAi groups displayed denser, more contiguous neocortical bone bridging the defect margins (Figure 6F). Masson's trichrome staining further revealed enhanced collagen matrix deposition and mineralization in TEAi‐treated defects, consistent with advanced osteoid maturation (Figure S7B). Immunohistochemical (IHC) analysis of osteopontin (OPN), a marker of osteoblast activity, showed significantly higher expression in TEAi groups, corroborating the pro‐osteogenic effects of TEAi (Figure S7C, D).

Notably, PDLSCs expressing methylation‐deficient TEAD3 mutants (R55K/ΔL1) in combination with TEAi exhibited the most pronounced regenerative outcomes, surpassing both wild‐type TEAD3+TEAi and untreated controls (Figure 6B–F and Figure S7B–D). This synergistic enhancement suggests that disrupting R55 methylation destabilizes repressive TEAD3 complexes in vivo, rendering them more susceptible to TEAi‐mediated disassembly. Consequently, liberated RUNX2 and other osteogenic factors likely drive accelerated bone matrix synthesis and remodeling.

These findings establish TEAi as a potent enhancer of PDLSC‐mediated bone regeneration, capable of improving both the quality (microarchitecture) and quantity (bone volume) of repaired tissue. The fact that its efficacy was most pronounced in the context of methylation‐deficient, hyper‐repressive TEAD3 mutants is consistent with our in vitro mechanistic model and underscores the therapeutic promise of targeting this axis. Future studies should explore whether systemic delivery of TEAi or localized modulation of TEAD methylation can achieve similar regenerative outcomes in larger animal models or human clinical settings.

## 3 Discussion

### 3.1 Post‐Translational Regulation of Osteogenesis: Expanding the Role of Arginine Methylation

Post‐translational modifications (PTMs) are pivotal regulators of protein activity and function, enabling dynamic cellular responses to developmental and environmental cues [30]. Our prior work has established that PTMs, including phosphorylation and ubiquitination, critically govern MSC lineage commitment [31, 32, 33, 34]. Here, we extend this paradigm by demonstrating that arginine methylation undergoes global upregulation during osteogenic differentiation, implicating it as a key regulatory mechanism. This observation aligns with emerging evidence that PRMTs modulate bone remodeling through diverse pathways. For instance, PRMT1 and PRMT7 enhance bone morphogenetic protein (BMP)‐driven osteogenesis, while PRMT5 inhibition promotes osteoblast differentiation [35, 36, 37, 38]. Notably, PRMT6 exhibits dual roles: it drives osteoclast differentiation via glycolytic reprogramming [39] and, as we reveal here, methylates TEAD3 at R55 to suppress osteogenesis. These findings highlight PRMT6 as a context‐dependent regulator, where its functional duality may stem from cell‐specific substrates (e.g., RANKL in osteoclasts vs. TEAD3 in osteoblasts) and interactions with distinct signaling hubs [40, 41, 42]. Our findings show that TEAD3 is a substrate for multiple PRMTs in vitro, revealing a previously unappreciated layer of network‐like regulation. This multiplexed enzymatic input likely confers robustness, redundancy, and signal‐integration capacity to the system in vivo. Rather than a single on‐off switch, R55 methylation appears to be a dynamic node, where the collective activity of context‐specific PRMTs fine‐tunes TEAD3's dimerization state and repressive function in response to diverse differentiation cues.

### 3.2 TEAD3 Methylation: a Non‐Canonical Hippo Pathway Regulatory Node

By identifying PRMT6 as one of the methyltransferases responsible for TEAD3‐R55 methylation, we uncover a novel regulatory axis within the Hippo signaling network. While canonical Hippo signaling converges on YAP/TAZ to drive TEAD‐mediated transcription, our data reveal a YAP/TAZ‐independent mechanism wherein R55 methylation governs TEAD3 homodimerization. Methylation loss (R55K) stabilizes TEAD3 dimers, sequestering RUNX2 into transcriptionally inert condensates and potently suppressing osteogenesis. This divergence from YAP/TAZ signaling underscores TEAD3's broader role in transcriptional regulation, extending beyond its canonical partnership with Hippo effectors.

### 3.3 Therapeutic Implications of TEAi: from Mechanism to Translation

The development of TEAi, a peptide targeting the TEA domain of TEAD3, represents a significant mechanistic and therapeutic advance. In this study, TEAi primarily served as a powerful molecular tool to validate our mechanistic model. It competitively binds to the TEA domain, disrupting TEAD3 homodimerization, liberating RUNX2 from inhibitory condensates, and thereby rescuing osteogenic differentiation in vitro. Crucially, its heightened efficacy against methylation‐deficient mutants (R55K/ΔL1) confirmed that pathological TEAD3 hyper‐dimerization creates a therapeutically exploitable vulnerability.

Our in vivo data further support the therapeutic potential of targeting this axis. The robust enhancement of bone regeneration by TEAi in a rat calvarial defect model demonstrates promising functional efficacy. However, we acknowledge that translating TEAi from a validated tool into a clinically viable therapy will require substantial future optimization. Key challenges include improving its in vivo stability, pharmacokinetic profile, and developing effective local delivery strategies.

In parallel, our work also reveals the challenges of an alternative therapeutic strategy: modulating the upstream methyltransferase activity. As shown in Figure S2, achieving a meaningful reduction in TEAD3 methylation in vivo would likely require concurrent inhibition of multiple PRMTs. Such combined inhibition, however, induced severe cytotoxicity, highlighting a significant translational hurdle for the use of broad‐spectrum PRMT inhibitors in regenerative contexts where progenitor cell survival is paramount. Therefore, direct targeting of the TEAD3‐RUNX2 interface with molecules like TEAi emerges as a more specific and potentially safer therapeutic avenue compared to upstream enzymatic inhibition.

### 3.4 Limitations and Future Directions

Our findings, while establishing a novel regulatory axis in PDLSC osteogenesis, also highlight several limitations and exciting avenues for future research.

First, while our in vivo experiment demonstrates compelling functional efficacy that TEAi robustly enhances bone regeneration, with effects magnified in the context of methylation‐deficient TEAD3 mutants, it provides phenotypic rather than direct molecular validation of the proposed condensate‐dissolution mechanism within the healing tissue. Future studies employing techniques such as in situ proximity ligation assays or spatial transcriptomics on regenerated bone sections could directly visualize TEAD3 condensate dynamics and RUNX2 liberation in vivo.

Second, several intriguing mechanistic observations warrant deeper investigation. The structural basis of how TEAi interacts with the TEA domain remains unclear; techniques like cryo‐EM or crystallography could clarify this binding interface and inform the design of more specific inhibitors. Additionally, the phenomenon of TEAi‐induced TEAD3 nuclear export, while consistent with our model of complex disruption, remains mechanistically unexplored. It may involve masking of a constitutive nuclear localization signal within the TEA domain [43] or interference with nuclear anchoring, hypotheses that future studies using exportin inhibitors like Leptomycin B or detailed mapping of shuttling signals could test. Furthermore, while we identify PRMT6 as one of several enzymes capable of methylating TEAD3 in vitro, the upstream physiological signals that regulate this specific PRMT network (e.g., metabolic stress, mechanical loading) and its crosstalk with other osteogenic pathways remain to be defined.

Third, from a translational perspective, our work delineates two strategic paths with distinct challenges. Developing TEAi (or its mimetics) into a viable therapy requires overcoming peptide‐specific hurdles such as in vivo stability, optimal local delivery, and long‐term safety assessment, steps best addressed in large‐animal models. Conversely, the alternative strategy of modulating the upstream methylation node via PRMT inhibitors is complicated by the functional redundancy among PRMTs and the significant cytotoxicity we observed upon combined inhibition, highlighting a key challenge for its regenerative application.

Finally, the broader pathological implications of a dysregulated TEAD3 methylation state remain to be explored. Such a state could contribute to impaired bone healing in conditions like osteoporosis, diabetic osteopathy, or aged microenvironments. Validating this hypothesis in patient‐derived MSCs and clinical specimens will be crucial for understanding the translational relevance of this axis.

Addressing these limitations through the outlined future work will be essential to advance these mechanistic insights toward clinical impact.

## 4 Conclusion

This work redefines TEAD3 as a PTM‐regulated hub integrating Hippo‐independent signals with osteogenic transcription. By elucidating the PRMT‐TEAD3‐RUNX2 axis and validating TEAi's therapeutic potential, we provide a roadmap for targeting methylation‐dependent transcriptional condensates in skeletal regeneration. Addressing the outlined limitations will be critical to advancing these insights toward clinical impact.

## 5 Materials and Methods

### 5.1 Cell Culture and Differentiation

This study was ethically reviewed and approved by the Medical Ethics Committee of the Hospital of Stomatology Tianjin Medical University, confirming compliance with all relevant ethical regulations and standards (Ethics Approval Number: TMUhMEC20230209). The study samples were sourced from patients aged 14 to 45 years with no history of periodontitis and in good oral health. The teeth selected were intact premolars or third molars that were extracted due to orthodontic treatment, prosthetic needs, or surgical reasons, with all specimens being non‐carious. All donors were systemically healthy and free of periodontal disease (Table S1). The periodontal ligament in the middle of the root was scraped off and digested for 30 min with a 1:1 digestion solution of collagenase and dispase enzyme. Newly prepared α‐MEM medium containing 10% fetal bovine serum (FBS) and 1% penicillin/streptomycin was used for resuspension precipitation, and the mixture was added to 25 cm^2^ culture flasks containing 4–5 mL of α‐MEM medium after the rotation at 1000 rpm/min for 4 min. The culture medium was refreshed at 3‐day intervals until 70–80% confluency was achieved. PDLSCs between passages 3 to 5 were selected for further experiments. For osteogenic induction, PDLSCs were maintained in a differentiation medium supplemented with 10 mm β‐glycerophosphate, 50 mg/L ascorbic acid, and 10 nm dexamethasone. For adipogenic induction, PDLSCs were maintained in a differentiation medium supplemented with 0.5 mm 3‐isobutyl‐1‐methylxanthine, 0.5 µm hydrocortisone, 60 µm indomethacin, and 10 µg/mL insulin.

### 5.2 Transfection and Lentiviral Infection

Human embryonic kidney 293T cells were maintained in DMEM containing 10% FBS at 37°C under 5% CO_2_. According to the manufacturer's protocol, Lipofectamine (Invitrogen) was used for plasmid transfection in 293T cells. Supernatants containing recombinant pLKO.1 (for knockdown) or pLvx (for overexpression) plasmid viruses were packaged in 293T cells. Then, lentivirus‐mediated knockdown or overexpression of PDLSCs was established through lentiviral infection and puromycin (2 µg/ml) selection. All primer sequences for overexpression and shRNA sequences are shown in the Tables S2 and S3.

### 5.3 Quantitative Real‐Time PCR

Total RNA was extracted from PDLSCs using TRIzol reagent (GenStar, P118‐05). cDNA was synthesized using oligo(dT) primers and reverse transcriptase according to the manufacturer's protocol (Thermoscientific). Quantitative RT‐PCR was performed using SYBR qPCR Master Mix (Vazyme, Q711‐02) according to the manufacturer's instructions. Each reaction was repeated at least three times. The expression results were normalized to RP0. Primer sequences for specific genes are shown in Supplementary Table S4.

### 5.4 Antibodies and Western Blot Analysis

The antibodies used in this research were as follows: the housekeeping protein glyceraldehyde phosphate dehydrogenase (ABclonal, AC001), GFP (ABclonal, AE078), Flag (Abclonal, AE092), TEAD3 (absin, abs117904), Myc (proteintech, 60003‐2‐Ig), HA (proteintech, 51064‐2‐AP), CtBP2 (ABclonal, A0463), TAZ (Cell Signaling Technology, D3I6D), and HRP Goat Anti‐Rabbit IgG (H+L) (ABclonal, AS014). PDLSCs and 293T were lysed with cell lysis buffer (E125‐01; GenStar) containing protease inhibitor cocktail (1:100; MedChemExpress) that was cooled to 4°C in advance to extract the protein. The lysate was rotated at 4 °C for 30 min, followed by boiling in SDS loading buffer at 95 °C for 15 min. Western blot analysis was conducted using a standard protocol.

### 5.5 Immunoprecipitation

The 293T cells were washed with cold PBS 36 to 48 h after PEI transfection. Then, the cells were lysed with cell lysis buffer (E125‐01; GenStar) containing protease inhibitor cocktail (1:100; MedChemExpress) that was cooled to 4°C in advance to extract the protein. The lysates were subjected to immunoprecipitation with anti‐Flag‐M2 magnetic beads (M8823, Sigma‐Aldrich) at 4°C overnight. Then the M2 beads were washed with cell lysate and detected with specific antibodies by Western blot.

### 5.6 Immunofluorescence

Cells were seeded on coverslips in 12‐well plates and cultured until the desired confluence was reached. Following fixation in 4% paraformaldehyde for 10 min at room temperature, the samples were washed three times with PBS. Permeabilization was carried out using 0.5% Triton X‐100 for 15 min, after which the cells were blocked with 5% BSA in PBS for 1 h. Subsequently, the samples were incubated with primary antibody overnight at 4 °C. After three additional PBS washes, FITC‐ or TRITC‐conjugated secondary antibodies were applied and incubated at 37 °C for 1 h. The coverslips were stained with DAPI and mounted. Then, the immunofluorescence images were captured by a confocal laser scanning microscope (Carl Zeiss LSM800, Germany).

### 5.7 In Vitro Methylation

In vitro PRMT‐mediated methylation assays were conducted as previously described [44]. Briefly, 1.5 µg of His‐tagged TEAD (wild‐type and R55K mutant) was incubated with 0.75 µg of GST‐PRMT1‐7, respectively, and 1.65 µCi of [methyl‐^3^H]‐S‐adenosyl methionine (SAM) in a 25 mm Tris–HCl buffer (pH 8.0) at 37°C for 8 h. The reaction was terminated by adding sodium dodecyl sulfate (SDS) sample buffer. Liquid scintillation counting was used to determine the amounts of radioactivity.

### 5.8 Animal Procedures

The animal experiment in this study was ethically reviewed and approved by the Medical Ethics Committee of the Institute of Radiation Medicine, Chinese Academy of Medical Sciences, and Peking Union Medical College (Ethics Approval Number: IRM/2‐IACUC‐2505‐004). In this research, 6‐8‐week‐old male Sprague‐Dawley (SD) rats purchased from Charles River were used for in vivo animal experiments. After 2 weeks of adaptive feeding, a calvarial bone defect model was performed, with 5 rats in each group. A full‐thickness bone defect with a diameter of 5 mm was created bilaterally on the calvarial bone of each rat, followed by the implantation of differently treated PDLSCs. The cells were delivered using Pluronic F‐127 hydrogel (Sigma, P2443) and gelatin sponge (Jiangxi Xiangen Medical Technology Department Co., Ltd.) as scaffolds. Preparation of the Pluronic F‐127 Hydrogel Loaded with PDLSCs: Pluronic F‐127 powder (5 g) was weighed into a 50 mL centrifuge tube, and PBS was added to a final volume of 25 mL. Pluronic F‐127 exists in a liquid state at 4°C. The mixture was stirred thoroughly at 4°C until homogeneous. The solution was poured into a culture dish and sterilized by exposure to ultraviolet light overnight. Upon completion of sterilization, the solution was aliquoted and stored. On the day of surgery, the prepared cells were trypsinized, centrifuged, and washed once with PBS. After discarding the supernatant, the cell was resuspended in pre‐chilled Pluronic F‐127 solution (4°C) in a centrifuge tube, which was kept on ice until use. The cell suspension density was adjusted to 1.5 × 10^6^ cells/mL. Immediately before application, the cell‐Pluronic F‐127 mixture was converted into a gel state by heating in a 37°C water bath and then implanted into the bone defect site. Preparation of the Gelatin Sponge Loaded with PDLSCs: Under sterile conditions in a laminar flow hood, gelatin sponges were cut into 2 mm × 2 mm × 2 mm cubes and placed into individual wells of a 96‐well plate. A 0.75 mg/L poly‐D‐lysine (PLL) (MCD5790, MesGen Biotechnology) solution was then added dropwise onto each sponge. The plate was incubated overnight at 37°C. Following incubation, the PLL solution was removed, and the sponges were washed three times with PBS and once with osteogenic induction medium. Subsequently, the sponges were soaked overnight in the osteogenic induction medium. After removing the original medium, the prepared cell suspension (cells resuspended in osteogenic induction medium) was added dropwise to the pre‐treated gelatin sponges. The cells were cultured for 3 days prior to experimentation. Then, the placement method of PDLSCs and scaffolds followed previously published literature [29]. After 12 weeks, samples were collected, and the bone defect repair was evaluated using Micro‐CT, HE staining, Masson staining, and IHC staining methods.

### 5.9 Statistical Analysis

All data were statistically analyzed by GraphPad Prism 9 software. All data were obtained by independent repeated experiments more than three times, expressed as mean ± standard deviation. One‐way ANOVA was used for multiple groups of samples, and a two‐tailed Student's *t*‐test was used between two groups of independent samples. *P* < 0.05 was considered statistically significant.

## Author Contributions

L.C. and R.H. contributed to the experiment design, experiment operation, data collection, statistical analyses, and manuscript writing. H.X. and Q.L. contributed to data analysis. R.H. and S.T. contributed to the animal experiment. L.C. contributed to conceptualizing the study, designed the experiments. X.W. and D.L. revised the manuscript, provided supervision and guidance for this study, and offered financial support.

## Conflicts of Interest

The authors declare no conflict of interest.

## Supporting information




**Supporting File 1**: advs73660‐sup‐0001‐SuppMat.pdf.

**Supporting File 2**: advs73660‐sup‐0002‐Supplementary Tables‐R1.docx.

## Data Availability

The data that support the findings of this study are available from the corresponding author upon reasonable request.
