# Supplementary material for: Arginine Methylation Antagonizes TEAD3‐Mediated Repression to Promote Osteogenic Differentiation by Disrupting RUNX2‐Sequestrating Condensates
Source: Adv Sci (Weinh). 2026 Jan 20;13(16):e18597. doi: 10.1002/advs.202518597 (PMC13042931; doi:10.1002/advs.202518597)
Supplement: Supplementary file 1 — Supporting File 1: advs73660‐sup‐0001‐SuppMat.pdf. [file ADVS-13-e18597-s001.pdf]

Figure S1

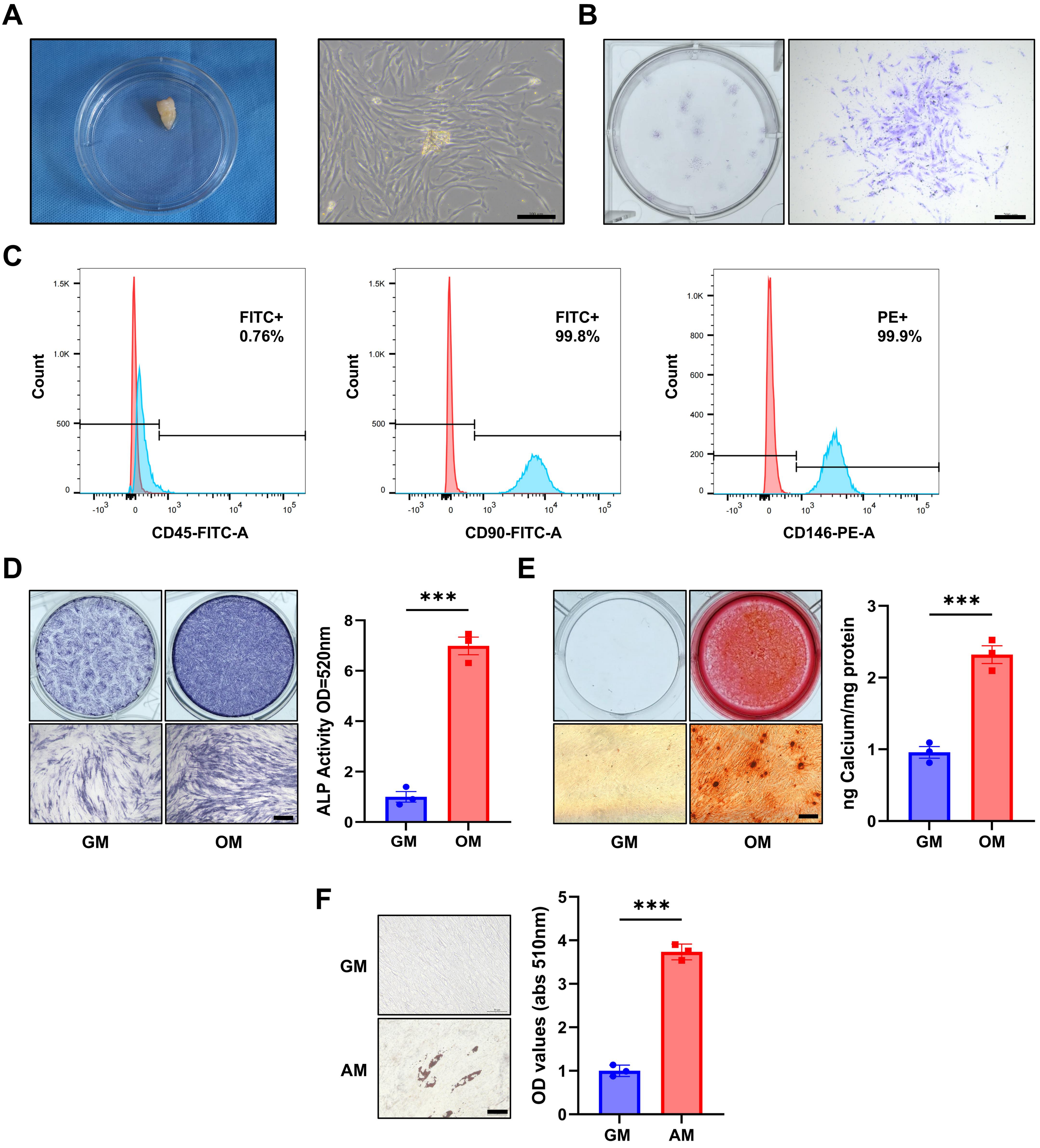

Figure S1

(A) PDLSCs were isolated from heathy human periodontal ligament. Scale bar, 100 μm. (B) The clone formation of PDLSCs. Scale bar, 200 μm. (C) Flow cytometry for the surface markers of PDLSCs. (D) ALP staining and ALP activity quantification at 7 days of osteogenic differentiation. GM: Growth Medium. OM: Osteogenic Differentiation Medium. Scale bar, 200 μm. (E) Alizarin red staining and quantitative analysis of calcium ion concentration at 14 days of osteogenic differentiation. Scale bar, 100 μm. (F) Oil red O staining and lipid droplet quantification at 21 days of adipogenic differentiation. AM: Adipogenic Differentiation Medium. Scale bar, 50 μm. ***P < 0.001. All data are expressed as means ± SD (n = 3).

Figure S2

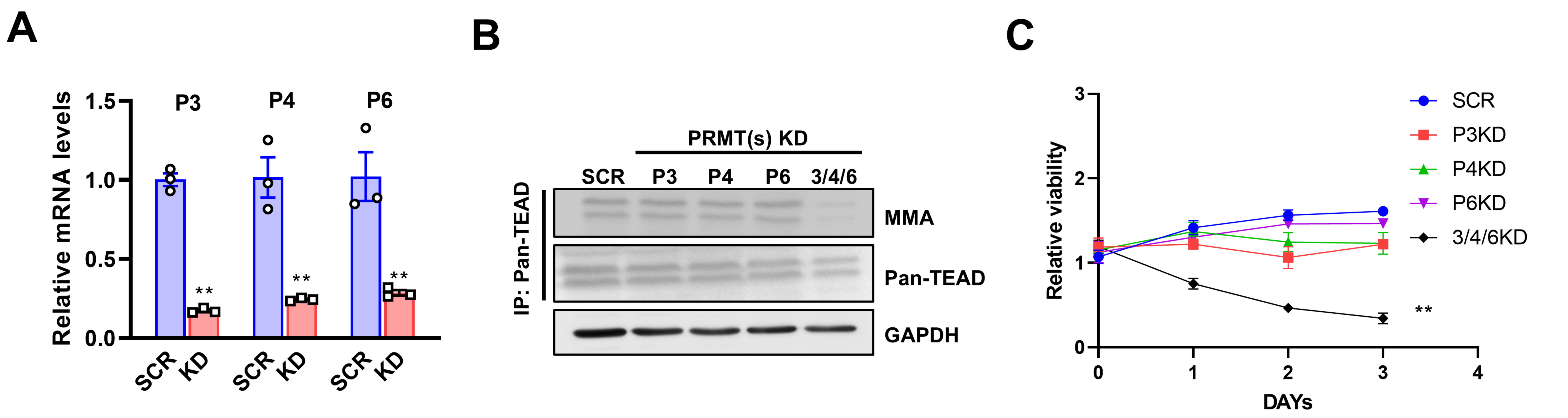

Figure S2

(A) Knockdown efficiency of PRMTs was measured by RT-qPCR. $**P < 0.01$. (B) The methylation level of pan-TEAD was analyzed by Co-IP. (C) The impact of PRMTs on the viability of PDLSCs. $**P < 0.01$.

Figure S3

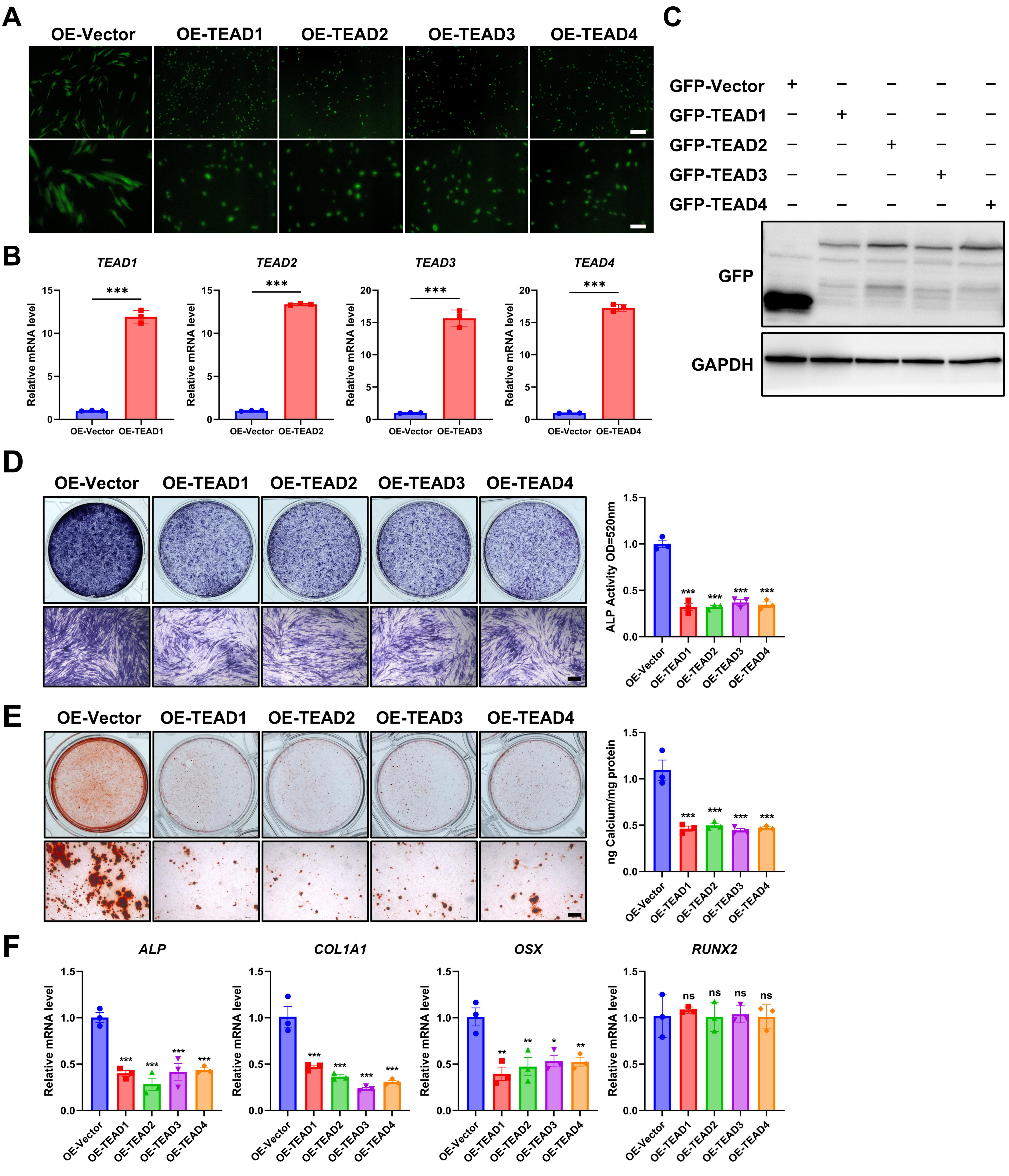
A
OE-Vector
OE-TEAD1
OE-TEAD2
OE-TEAD3
OE-TEAD4
B
TEAD1
TEAD2
TEAD3
TEAD4
Relative mRNA level
***
OE-Vector
OE-TEAD1
OE-TEAD2
OE-TEAD3
OE-TEAD4
C
GFP-Vector + − − − −
GFP-TEAD1 − + − − −
GFP-TEAD2 − − + − −
GFP-TEAD3 − − − + −
GFP-TEAD4 − − − − +
GFP
GAPDH
D
ALP Activity OD=520nm
E
ng Calcium/mg protein
F
ALP
COL1A1
OSX
RUNX2
ns
**
*

Figure S3

(A) IF analysis for detection of overexpressed TEAD1-4 with GFP tag. Scale bar, 200 μm (top) and 100 μm (bottom). OE-Vector: overexpression control group. OE-TEAD1: TEAD1 overexpression group. OE-TEAD2: TEAD2 overexpression group. OE-TEAD3: TEAD3 overexpression group. OE-TEAD4: TEAD4 overexpression group. (B) Overexpression efficiency of TEAD in PDLSCs was measured by RT-qPCR. (C) The detection of overexpressed TEAD1-4 with GFP tag. (D) ALP staining and ALP activity quantification at 7 days of osteogenic differentiation. Scale bar, 200 μm. (E) Alizarin red staining and quantitative analysis of calcium ion concentration at 14 days of osteogenic differentiation. Scale bar, 200 μm. (F) Relative mRNA levels of osteogenic marker genes were quantified by RT-qPCR at 7 days of osteogenic differentiation. *$P < 0.05$, **$P < 0.01$, ***$P < 0.001$, ns: $P > 0.05$ vs. OE-Vector. All data are expressed as means ± SD (n = 3).

Figure S4

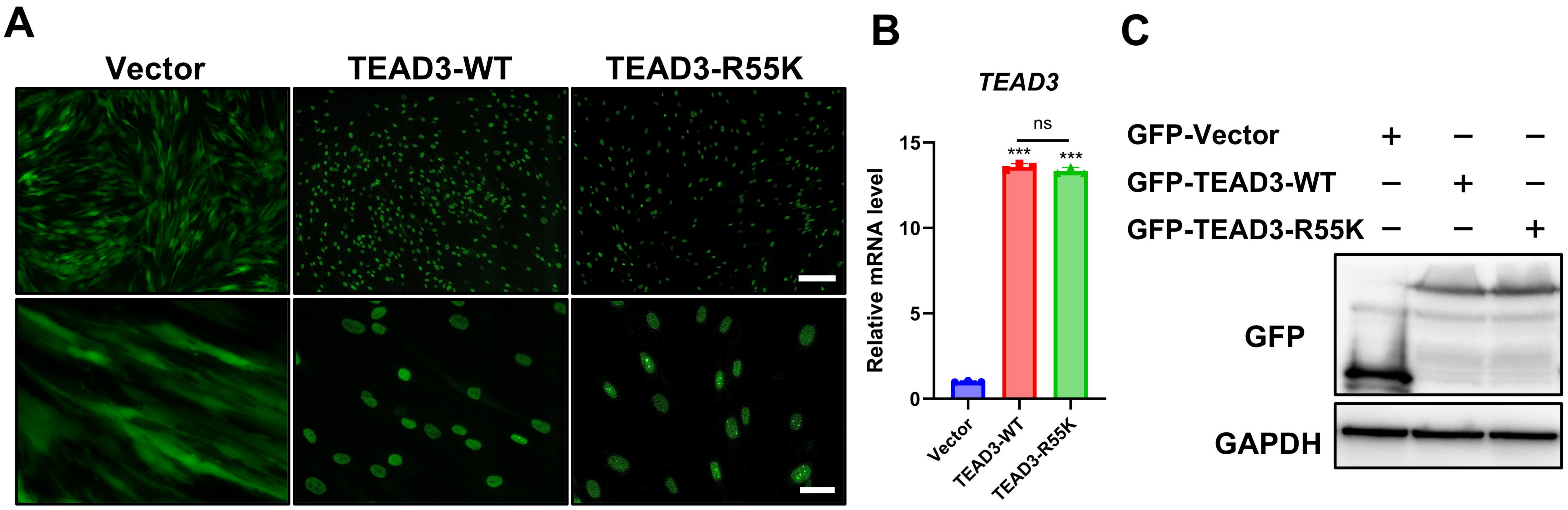

Figure S4

(A) IF analysis for detection of overexpressed TEAD WT and R55K mutant with GFP tag. Scale bar, 200 μm (top) and 50 μm (bottom). (B) Overexpression efficiency of TEAD WT and R55K mutant in PDLSCs was measured by RT-qPCR. ****P* < 0.001 vs. Vector, ns: *P* > 0.05. All data are expressed as means ± SD (n = 3). (C) The detection of overexpressed TEAD WT and R55K mutant with GFP tag.

Figure S5

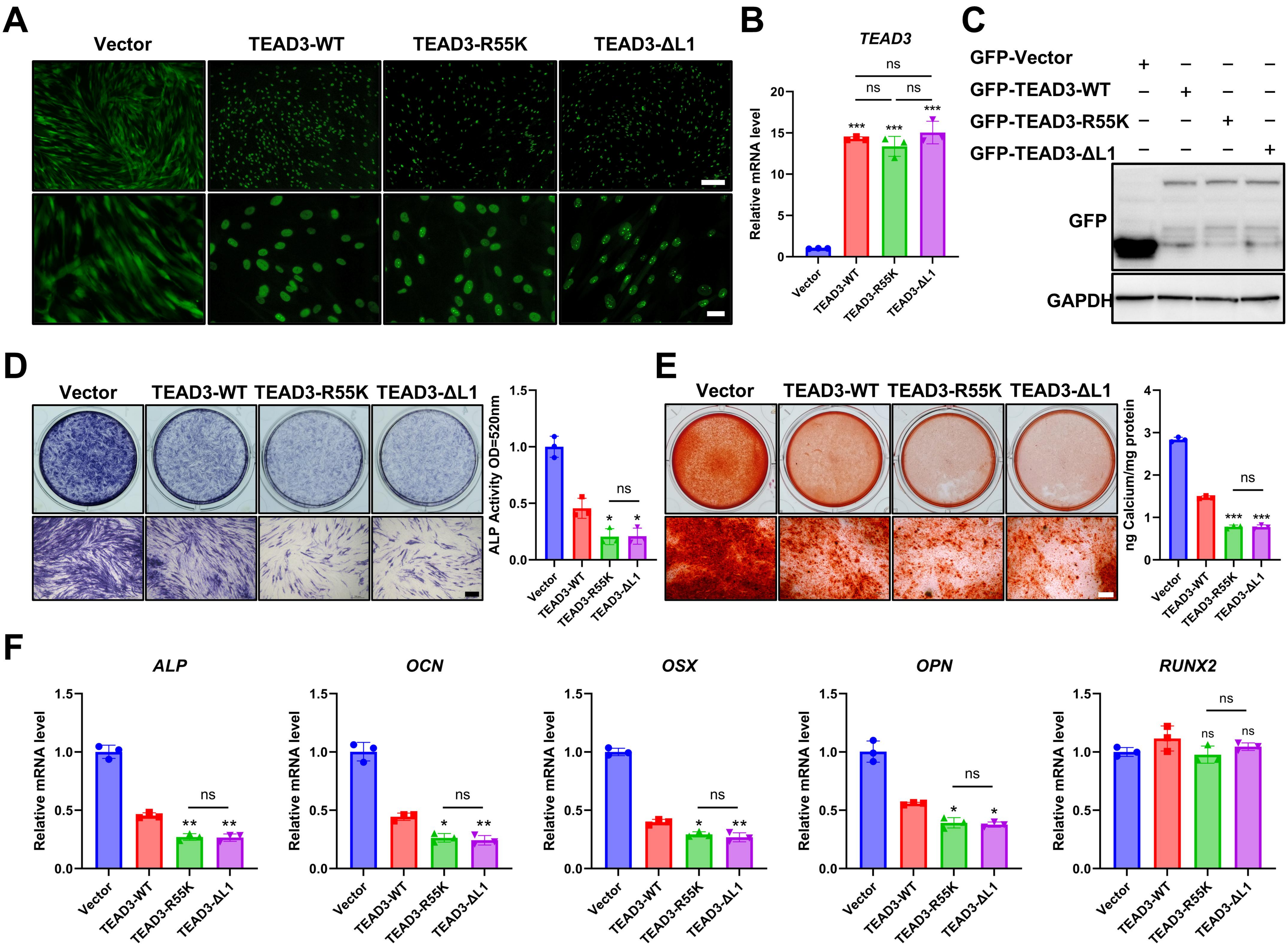

Figure S5

(A) IF analysis for detection of overexpressed TEAD WT, R55K and ΔL1 mutant with GFP tag. Scale bar, 200 μm (top) and 40 μm (bottom). (B) Overexpression efficiency of TEAD WT, R55K and ΔL1 mutant in PDLSCs was measured by RT-qPCR. ***$P < 0.001$ vs. Vector, ns: $P > 0.05$. (C) The detection of overexpressed TEAD WT, R55K and ΔL1 mutant with GFP tag. (D) ALP staining and ALP activity quantification at 7 days of osteogenic differentiation. Scale bar, 200 μm. (E) Alizarin red staining and quantitative analysis of calcium ion concentration at 14 days of osteogenic differentiation. Scale bar, 200 μm. (F) Relative mRNA levels of osteogenic marker genes were quantified by RT-qPCR at 7 days of osteogenic differentiation. *$P < 0.05$, **$P < 0.01$, ***$P < 0.001$ vs. Vector, ns: $P > 0.05$. All data are expressed as means ± SD (n = 3).

Figure S6

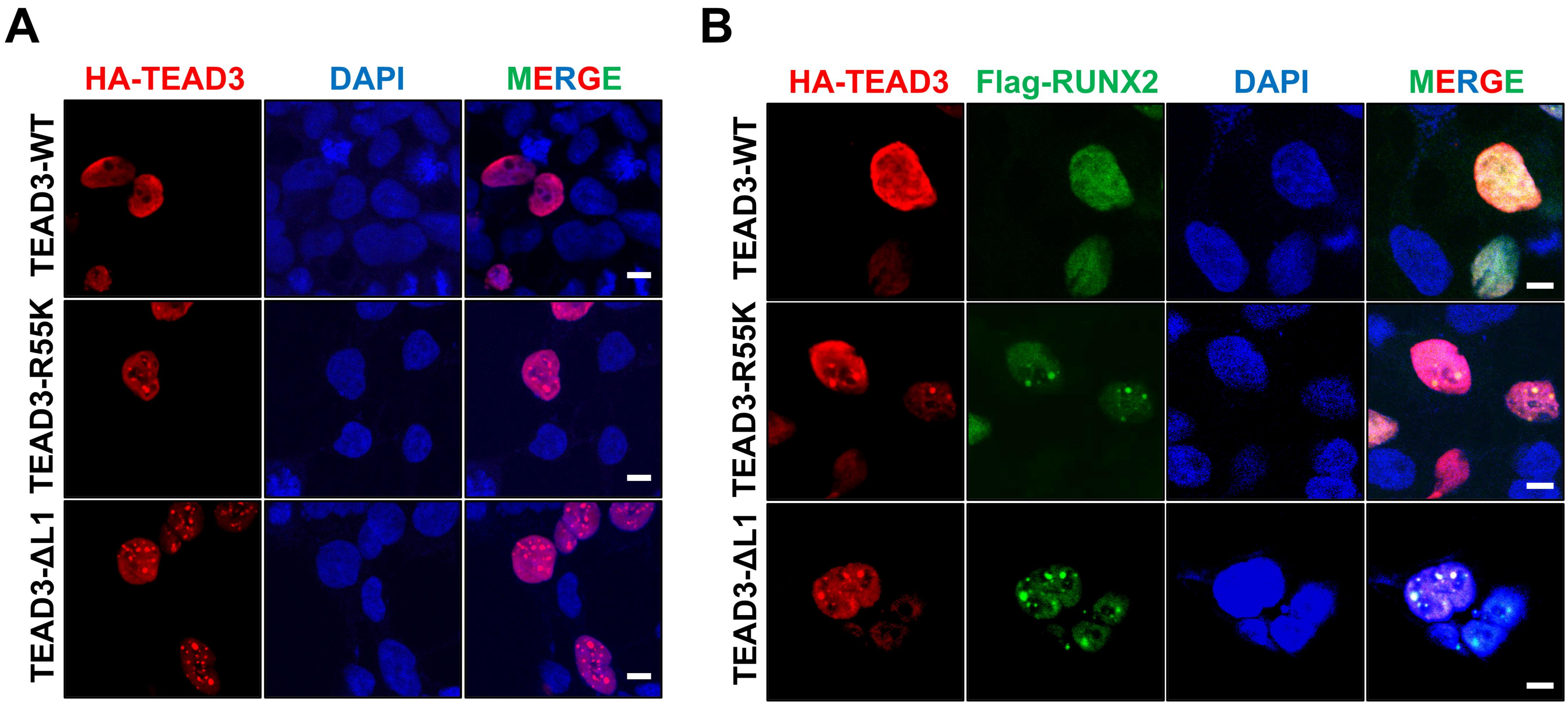

Figure S6

(A) IF analysis to TEAD WT, R55K and ΔL1 with Flag tag. Scale bar, 10 μm. (B) IF analysis of Flag tagged TEAD and its mutants with RUNX2. Scale bar, 10 μm.

Figure S7

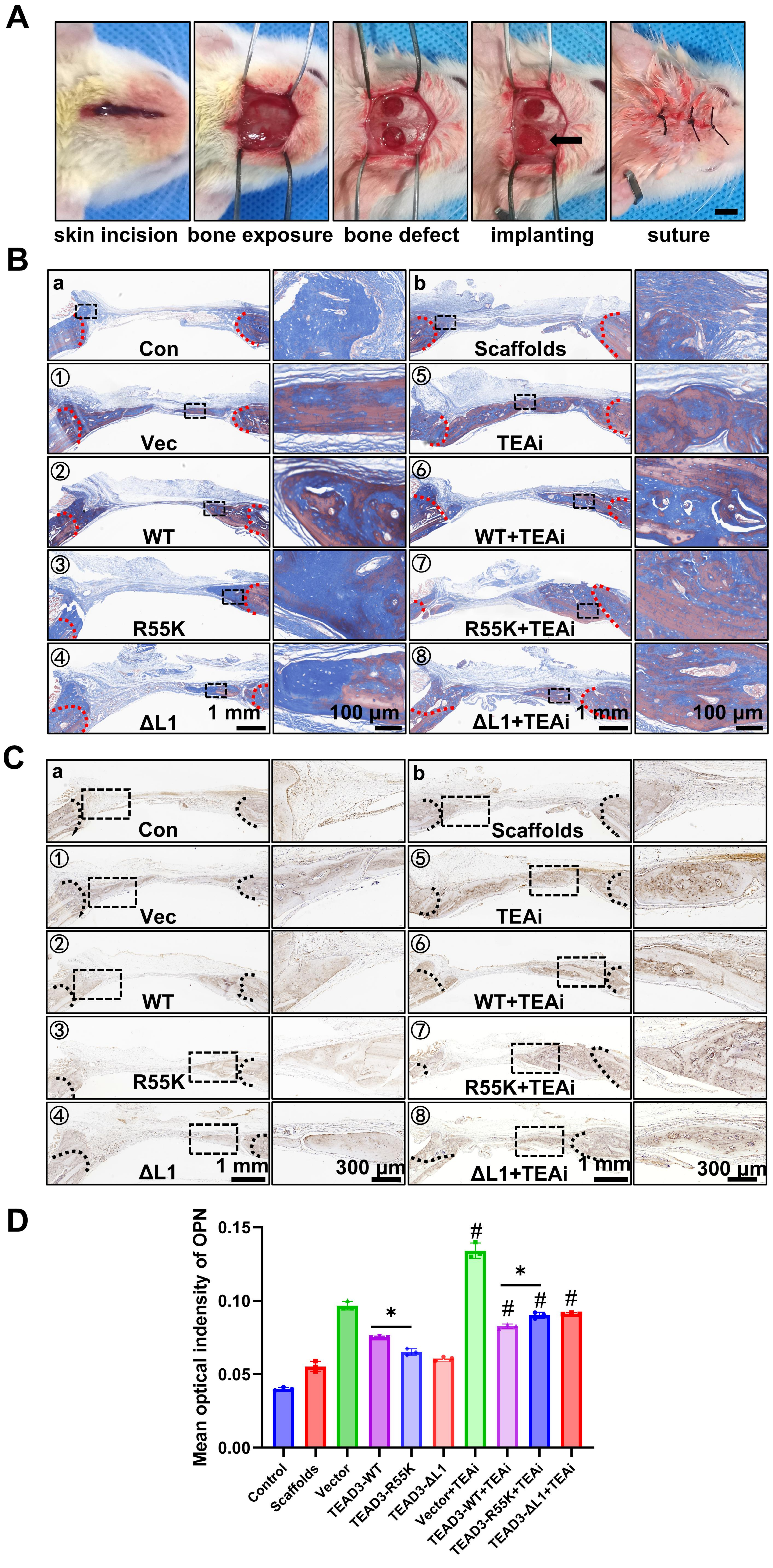

Figure S7

(A) Model establishment of calvarial bone defect of rat. Black arrow: Implanted PDLSCs and scaffolds. Scale bar, 5 mm. (B) Masson staining to detect the new bone mineralization in bone defect areas. (C) The IHC staining of OPN in bone defect areas. (D) Quantitative analysis of IHC staining of OPN in bone defect areas. $^{\#}P < 0.05$ vs. corresponding control group without TEAi. $^{*}P < 0.05$. All data are expressed as means ± SD (n = 3).
