# Supplementary material for: Arginine Methylation Antagonizes TEAD3‐Mediated Repression to Promote Osteogenic Differentiation by Disrupting RUNX2‐Sequestrating Condensates
Source: Adv Sci (Weinh). 2026 Jan 20;13(16):e18597. doi: 10.1002/advs.202518597 (PMC13042931; doi:10.1002/advs.202518597)
Supplement: Supplementary file 2 — Supporting File 2: advs73660‐sup‐0002‐Supplementary Tables‐R1.docx. [file ADVS-13-e18597-s002.docx]

**Table S1.** **Donor Information of PDLSCs**

| **Donor ID** | **Gender (M/F)** | **Age**  **(Years Old)** | **General Health** | **Reason for Tooth Extraction** | **Tooth Number** |
| --- | --- | --- | --- | --- | --- |
| 001 | M | 14 | Good | Orthodontics | 24 |
| 002 | M | 15 | Good | Orthodontics | 15 |
| 003 | F | 23 | Good | Third Molars | 38 |

**Table S2. Sequences of primers for overexpress TEAD**

| **Genes** | **Sequence (5’-3’)** |
| --- | --- |
| OE-*TEAD1*-F | AAGGGGGGAGGAGGGGGATCCATGATTGAGCCCAGCAGCTGGAGC |
| OE-*TEAD1*-R | CTCCCCTACCCGGTAGAATTCTCAGTCCTTTACAAGCCTGTA |
| OE-*TEAD2*-F | AAGGGGGGAGGAGGGGGATCCATGGGGGAACCCCGGGCTGGG |
| OE-*TEAD2*-R | CTCCCCTACCCGGTAGAATTCTCAGTCCCTGACCAGGCGGTA |
| OE-*TEAD3*-F | AAGGGGGGAGGAGGGGGATCCATGATAGCGTCCAACAGCTGGAAC |
| OE-*TEAD3*-R | CTCCCCTACCCGGTAGAATTCCTAGTCTTTGACGAGCTTGTA |
| OE-*TEAD4*-F | AAGGGGGGAGGAGGGGGATCCATGTTGGAGGGCACGGCCGGCACC |
| OE-*TEAD4*-R | CTCCCCTACCCGGTAGAATTCTCATTCTTTCACCAGCCTGTA |

**Table S3. Sequences of primers for knockdown TEAD and PRMTs**

| **Genes** | **Sequence (5’-3’)** |
| --- | --- |
| sh*TEAD1*-F | CCGGCCGATTTGTATACCGAATAAACTCGAGTTTATTCGGTATACAAATCGGTTTTTG |
| sh*TEAD1*-R | AATTCAAAAACCGATTTGTATACCGAATAAACTCGAGTTTATTCGGTATACAAATCGG |
| sh*TEAD2*-F | CCGGCCCGAAGGAAATCAAGGGAAACTCGAGTTTCCCTTGATTTCCTTCGGGTTTTTG |
| sh*TEAD2*-R | AATTCAAAAACCCGAAGGAAATCAAGGGAAACTCGAGTTTCCCTTGATTTCCTTCGGG |
| sh*TEAD3*-F | CCGGGAGTTGATTGCACGCTATATTCTCGAGAATATAGCGTGCAATCAACTCTTTTTG |
| sh*TEAD3*-R | AATTCAAAAAGAGTTGATTGCACGCTATATTCTCGAGAATATAGCGTGCAATCAACTC |
| sh*TEAD4*-F | CCGGGAGACAGAGTATGCTCGCTATCTCGAGATAGCGAGCATACTCTGTCTCTTTTTG |
| sh*TEAD4*-R | AATTCAAAAAGAGACAGAGTATGCTCGCTATCTCGAGATAGCGAGCATACTCTGTCTC |
| sh*PRMT3*-F | CCGGCAGCCTTGTAGCAGTGAGTGACTCGAGCAGCCTTGTAGCAGTGAGTGATTTTTG |
| sh*PRMT3*-R | AATTCAAAAACAGCCTTGTAGCAGTGAGTGACTCGAGCAGCCTTGTAGCAGTGAGTGA |
| sh*PRMT4*-F | CCGGGCAAGCAGTCCTTCATCATCACTCGAGTGATGATGAAGGACTGCTTGCTTTTTG |
| sh*PRMT4*-R | AATTCAAAAAGCAAGCAGTCCTTCATCATCACTCGAGTGATGATGAAGGACTGCTTGC |
| sh*PRMT6*-F | CCGGGCCCAGTTTGAGATGCCTTATCTCGAGATAAGGCATCTCAAACTGGGCTTTTTG |
| sh*PRMT6*-R | AATTCAAAAAGCCCAGTTTGAGATGCCTTATCTCGAGATAAGGCATCTCAAACTGGGC |

**Table S4. Sequences of primers for RT-qPCR**

| **Genes** | **Sequence (5’-3’)** |
| --- | --- |
| *RP0*-F | TTCATTGTGGGAGCAGAC |
| *RP0*-R | CAGCAGTTTCTCCAGAGC |
| *TEAD1*-F | ATGGAAAGGATGAGTGACTCTGC |
| *TEAD1*-R | TCCCACATGGTGGATAGATAGC |
| *TEAD2*-F | GCCTCCGAGAGCTATATGATCG |
| *TEAD2*-R | TCACTCCGTAGAAGCCACCA |
| *TEAD3*-F | TGGACCCTCTCAGGACATCAA |
| *TEAD3*-R | CCAGGGGCTCATAACTGCTG |
| *TEAD4*-F | GGACACTACTCTTACCGCATCC |
| *TEAD4*-R | TCAAAGACATAGGCAATGCACA |
| *ALP*-F | ACAAGCACTCCCACTTCATC |
| *ALP*-R | TTCAGCTCGTACTGCATGTC |
| *OCN*-F | CACTCCTCGCCCTATTGGC |
| *OCN*-R | CCCTCCTGCTTGGACACAAAG |
| *OPN*-F | CTCCATTGACTCGAACGACTC |
| *OPN*-R | CAGGTCTGCGAAACTTCTTAGAT |
| *RUNX2*-F | AGAAGGCACAGACAGAAGCTTGA |
| *RUNX2*-R | AGGAATGCGCCCTAAATCACT |
| *COL1A1*-F | GAGGGCCAAGACGAAGACATC |
| *COL1A1*-R | CAGATCACGTCATCGCACAAC |
| *OSX*-F | CAACTGGCTCTTCTGCGGCAAGAG |
| *OSX*-R | GCTGGTGTTTGCTCAGGTGGTC |
| *PRMT3*-F | GTACCCTTCTCATACCCCAATGG |
| *PRMT3*-R | GACGAGCAGGTTCTGACATCT |
| *PRMT4*-F | GGGCTACATGCTCTTCAACG |
| *PRMT4*-R | GTCCACTCCATGGAAAGATGG |
| *PRMT6*-F | TCTGGTTCCAGGTGACCTTC |
| *PRMT6*-R | AGGTAGAGGAGCGCCTGTTT |
